# Health-related quality of life outcomes at 1 and 5 years after a residential retreat promoting lifestyle modification for people with multiple sclerosis

**DOI:** 10.1007/s10072-012-0982-4

**Published:** 2012-02-25

**Authors:** Emily J. Hadgkiss, George A. Jelinek, Tracey J. Weiland, Greg Rumbold, Claire A. Mackinlay, Siegfried Gutbrod, Ian Gawler

**Affiliations:** 1 Emergency Practice Innovation Centre, St. Vincent’s Hospital, Fitzroy, P.O. Box 2900, Melbourne, VIC 3065 Australia; 2 Faculty of Medicine, Dentistry and Health Sciences, University of Melbourne, Melbourne, Australia; 3 The Gawler Foundation, Yarra Junction, VIC Australia

**Keywords:** Quality of life, MSQOL-54, Multiple sclerosis, Well-being, Lifestyle

## Abstract

There is a strong body of evidence that supports the use of non-drug therapies in the management of people with multiple sclerosis (MS). A 5-day residential retreat for people with MS in Victoria, Australia, promotes lifestyle modification within a patient-centred model of care. Analysis of the health-related quality of life (HRQOL) of the retreat participants was undertaken using the MSQOL-54, prior to attendance, 1 and 5 years after the retreat. 274 retreat participants (71%) completed baseline questionnaires. Despite the usually progressive nature of MS, the cohort demonstrated clinically and statistically significant improvements in HRQOL. One year after attending the retreat, median improvements of 11.3% were observed in the overall quality of life domain (*p* < 0.001); 18.6% in the physical health composite (*p* < 0.001); and 11.8% in the mental health composite (*p* < 0.001). In the subset of 165 who had reached the 5-year time-point, there was a 19.5% median improvement in overall quality of life (*p* < 0.001); 17.8% in the physical health composite (*p* < 0.001) and 22.8% in the mental health composite (*p* < 0.001), compared to baseline. Attendance at a retreat promoting lifestyle modification for the integrated management of MS appears to have positive effects on short and medium-term HRQOL. Non-drug therapies should be considered as part of any comprehensive treatment plan for people with MS.

## Introduction

Multiple sclerosis (MS) is the most common progressive neurological disorder affecting young adults [1]. Women are twice as likely as men to be affected with MS, with an estimated incidence rate of 3.6 per 100,000 [2], varying considerably with geographic region. MS is thought to be immune-mediated, however, despite decades of research, the aetiology and factors that trigger relapses and influence disease progression continue to be debated [3]. Disease activity and clinical course are unpredictable, usually resulting in a broad range of symptoms and functional limitations, with depression and fatigue the most common [4]. Many patients report feelings of hopelessness and uncertainty, particularly if they are not actively engaged in decision-making about their care [5]. Additionally, MS can have a major impact on families due to increased role responsibilities, social isolation, employment challenges and financial hardship.

Current treatment options for MS focus mainly on pharmaceutical intervention. First line immune-modulating drugs such as beta interferon and glatiramer acetate reduce relapse rates by around 30%, show MRI evidence of reducing disease activity, and can help preserve cognition; however there remain concerns regarding the short- and long-term side effects of such drugs and whether they make a real difference to the progression of the illness [6–8]. Nonetheless, observational studies have suggested that the disease-modifying treatments may have increased the survival, with somewhat slower accumulation of disability, raising the possibility of a new natural history of the illness [9]. This may, however, to some extent reflect increased recognition of MS with better diagnostic techniques [9].

Comprehensive literature also exists examining the relationship between modifiable lifestyle and psychosocial factors and MS progression [10]. However, most clinicians adopt a drug-only approach to disease modification, despite calls for a more integrated approach [11].

Consumer demand for non-drug intervention is evident through the large proportion of people with MS using complementary and alternative medicines in addition to mainstream medical treatment [12]. Likewise, wellness programmes provide a supportive, informative environment well-suited to assist individuals in self-managing their diverse symptomatology. Despite this, there is limited literature evaluating the effectiveness of this type of approach to MS management. Two systematic reviews [13, 14] of wellness programmes discuss the importance of patient-centred, multidisciplinary approaches in the management of chronic illness. Both studies found that despite the reported benefit of wellness programmes, the existing literature is deficient and mainly consists of only short-term follow-up.

A live-in educational programme at The Gawler Foundation in Victoria, Australia, provided a unique opportunity to assess the impact of such modifiable lifestyle factors on MS outcomes. Due to the unpredictable nature of the illness, is it important to evaluate MS outcomes in the medium to long-term. To date, apart from our published findings of health-related quality of life (HRQOL) outcomes at one and two-and-a-half years after attending this programme [15], there have been no other studies examining the effect of a multimodal residential programme promoting lifestyle modification for people with MS. As we have continued to collect follow-up data from this cohort, we aimed to present results of analyses of the cohort at the 1 and 5 year marks post-retreat.

## Materials and methods

### Programme

Since April 2002, a 5-day live-in programme for people with MS has been operating two to three times a year at The Gawler Foundation in the Yarra Valley in Victoria, Australia. Funded by donations and course fees, this not-for-profit organisation is experienced with operating integrated wellness programmes for people living with chronic illness and can accommodate up to 36 people, including partners and carers of people with MS. The only other comparable programme is offered by the MS Society of Auckland and the North Shore in New Zealand, facilitated by one of the authors [16]. The MS programme has been designed in accordance with the available literature that supports the integrated management of MS with: a low-fat, plant-based diet; exercise; sunlight exposure; vitamin D and omega-3 supplementation; and meditation and stress reduction techniques (Box 1). The evidence supporting the programme interventions has been discussed elsewhere [10, 11, 15, 17–19]. In addition to provide evidence-based education about lifestyle modification, participants engage in daily meditation, group and individual counselling, gentle exercise, yoga and Qigong. The programme is taught in a dynamic, interactive way which allows the participants to share their experiences and ask questions. Beyond the programme, the facilitators encourage participants to develop the various modalities into an ongoing treatment regime with medical oversight by their usual healthcare providers.

**Box 1:** Retreat recommendations (adapted from Li et al. [15])

Diet and supplements
Plant-based wholefood diet plus fish; minimal saturated fat Omega-3 fatty acid supplements: 20 g a day (flaxseed or fish oil)
Vitamin D
Sunlight 15 min daily 3–5 times a week Vitamin D3 5,000 IU daily Aim for vitamin D level around 150 nmol/L
Meditation
30 min daily
Exercise
30 min 5 times a week
Medication
One of the disease-modifying drugs, beginning early in the illness if possible, if required Steroids for any distressing acute relapse

### Methods

Participants who enrolled in a MS retreat at The Gawler Foundation were invited to take part. In order to attend the retreat, participants must be over 18 years of age and speak English proficiently. Anyone who self-reported being diagnosed with MS by a neurologist was eligible for the study. Those providing consent completed a validated questionnaire, the Multiple Sclerosis Quality Of Life (MSQOL-54) [20], prior to attending the retreat, and again at 1-, 2.5- and 5-year time-points. The 2.5-year time-point was phased out in 2009 to reduce the burden of compliance. A funded research officer commenced oversight of the dataset in 2008. The research officer attempted to locate participants who had changed address and actively sought responses from participants. An online questionnaire was offered to participants for baseline and follow-up in addition to the existing paper-based version in February 2009. Only those who completed a baseline were invited to continue participation. In the early phase of the study clerical issues meant that some groups did not receive an invitation to participate at the 1-year time-point.

### Ethics

A detailed explanation of the research was given to all participants at every time-point and formal, written consent was obtained. It was emphasised that a decision to decline participation would not affect their experience or interactions with staff at The Gawler Foundation. Approval to conduct this study was granted by the Human Research Ethics Committee of the University of Melbourne (HREC ID: 0723028.1). All survey data were subjected to confidentiality and patient codes were only re-identified for the purpose of contacting participants for follow-up.

### Evaluation tool

Evaluation of the programme at the time-points was facilitated by patient self-report using the MSQOL-54, which has been adapted from the RAND 36-item health survey and has demonstrated sound psychometric properties [20]. Since the introduction of the MSQOL-54 in 1995, it has been widely used as an outcome measure and has been culturally and linguistically adapted for the purpose of international research [21–24]. In addition to the core health survey questions, the MSQOL-54 has been supplemented to include measures of health distress, sexual function, energy, pain and social function, thus giving rise to 14 scales. Table 1 shows the weighted sum of selected domains which determine physical and mental health composite scores. Like the individual scale scores, the composite scores range from 0 to 100, with higher scores indicating a better health outcome.

**Table 1:** Weighted sum of MSQOL 54 domains

Physical health composite		Mental health composite	
Domains	Weight	Domains	Weight
Physical function	0.17	Health distress	0.14
Health perceptions	0.17	Overall quality of life	0.18
Energy/fatigue	0.12	Emotional well-being	0.29
Role limitations due to physical problems	0.12	Role limitations due to emotional problems	0.24
Pain	0.11	Cognitive function	0.15
Sexual function	0.08		
Social function	0.12		
Health distress	0.11		

### Statistical analysis

As the data were not normally distributed (Kolmogorov–Smirnov statistic), the Friedman’s test and Wilcoxon Signed Ranks test were used to make comparisons between baseline, 1- and 5-year time-points and the Mann–Whitney *U* test to compare the cohort with those lost to follow-up. Medians were used as summary statistics. The evaluation of the 2.5-year surveys was published elsewhere [15] and as no new data were available for this time point, it was excluded from analysis. The time-points were assessed separately as few participants completed all three surveys. Prior to analysis of the data, the level of significance was defined as *p* < 0.05. All statistical analyses were carried out with PASW 18.0 software.

## Results

### Participation rates

A total of 394 participants attended a retreat for MS at The Gawler Foundation between April 2002 and August 2010. Seven were excluded from the study as they were not formally diagnosed with MS or had another neurological condition. All 387 eligible participants completed the full 5-day programme. Of those eligible to participate, 274 (71%) completed a baseline questionnaire, of whom 227 (83%) were female. Due to the predominant representation of women in the cohort, gender comparisons were not made. The smaller subset of participants who completed 5-year surveys is due to the limited number (*n* = 165) who had completed a baseline questionnaire and reached the 5-year time-point. A smaller number of people completed surveys at all three time-points (*n* = 47).

Participation rates vary between domains as they have been adjusted according to the scoring criteria set by Vickrey 1995 [20], which requires a minimum number of items to be completed to give rise to a domain score. Accordingly, there is variation in the response rates for individual domains at each time-point. Baseline overall quality of life was compared between those who only completed baseline and were subsequently lost to follow-up and those who completed baseline and at least one other time-point. There was no statistically significant difference in the physical health composite (*p* = 0.414) or mental health composite (*p* = 0.131) between these two groups when compared using the Mann–Whitney *U* test.

### Domain rank comparison baseline, 1 and 5 years

The Friedman test was conducted to compare results for each domain at all three time-points and detected significant differences in 9 of 14 domain scores and both mental and physical health composites (Table 2). As only 47 participants had completed surveys at all time-points, separate post hoc tests were run to compare baseline to 1-year results and baseline to 5-year results, which represented a larger proportion of the cohort.

**Table 2:** Comparison of mean ranks at baseline, one year and five year time points

Domain scores and composite scores	*n*	Mean rank			Chi-square	*p* value
		Baseline	1 year	5 year		
Physical function	47	1.89	2.13	1.98	1.653	0.438
Role limitations-physical	46	1.74	2.18	2.08	8.309	0.016
Role limitations-emotional	43	1.73	2.12	2.15	11.400	0.003
Pain	46	1.97	2.13	1.90	1.708	0.426
Emotional well-being	47	1.61	1.94	2.46	19.602	0.000
Energy	47	1.60	2.24	2.16	12.489	0.002
Health perceptions	46	1.55	2.08	2.37	17.815	0.000
Social function	43	1.77	2.01	2.22	5.729	0.057
Cognitive function	47	1.74	2.24	2.01	7.789	0.020
Health distress	45	1.44	2.23	2.32	23.346	0.000
Sexual function	42	2.11	2.05	1.85	1.942	0.379
Overall quality of life	47	1.51	2.18	2.31	20.808	0.000
Change in health	45	1.76	2.37	1.88	14.356	0.001
Satisfaction with sexual function	41	1.87	2.23	1.90	4.748	0.093
Physical health composite	37	1.57	2.30	2.14	10.865	0.004
Mental health composite	41	1.54	2.20	2.27	13.317	0.001

Differences assessed using Friedman test

*n* Number of participants

### 1-year time-point

**Fig. 1:**
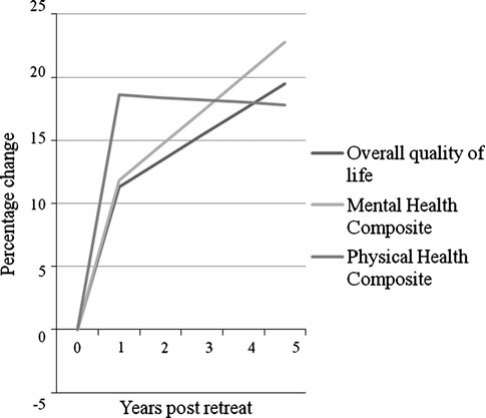
Percentage change in median MSQOL-54 composite and overall quality of life scores at baseline, 1 and 5 years after a residential retreat. *All differences represent significant improvement (*p* < 0.001) from baseline

Of the 270 participants sent invitations at the 1-year time-point, surveys were completed by 196 participants, for a 73% response rate. At 1 year, 13 of 14 domains and both composite scores showed positive median changes, demonstrating improvements in HRQOL. The median changes were significantly different from zero in 11 of these 13 domains and both physical and mental health composite scores. No change in median score was observed in role limitations due to emotional problems, although the Wilcoxon Signed Ranks test detected a significant difference (*p* = 0.002). Improvements for physical health and sexual function were not statistically significant. The greatest change over time was seen in the domains: role limitations due to physical problems; change in health; and satisfaction with sexual function. They all demonstrated a 50% (25 point) improvement 1 year after baseline. The smallest statistically significant improvement was observed for cognitive function, which increased by five points. At 1 year overall quality of life improved 11.3% (*p* < 0.001), physical health composite 18.6% (*p* < 0.001) and mental health composite 11.8% (*p* < 0.001) (Table 3; Fig. 1).

**Table 3:** Comparison of domain and composite scores at baseline and 1-year time-points

Domain	*n*	Baseline	1 year	Domain point difference	% median difference	*Z* score	*p* value
		Median (IQR)					
Physical health	190	75.0 (40.0–90.0)	80.0 (45.0–95.0)	5.0	6.7	−1.401	0.161
Role limitations-physical	194	50.0 (0.0–100.0)	75.00 (0.00–100.00)	25.0	50.0	−3.791	<0.001
Role limitations- emotional	185	100.0 (33.3–100.0)	100.0 (66.7–100.0)	0.0	0.0	−3.108	0.002
Pain	190	85.8 (70.0–100.0)	93.3 (76.7–100.0)	7.5	8.7	−3.044	0.002
Emotional well-being	196	72.0 (60.0–84.0)	80.0 (68.0–88.0)	8.0	11.1	−4.696	<0.001
Energy	196	48.0 (32.0–67.0)	60.0 (40.0–72.0)	12.0	25.0	−4.658	<0.001
Health perceptions	191	60.0 (45.0–75.0)	70.0 (55.0–85.0)	10.0	16.7	−5.658	<0.001
Social function	183	75.0 (58.3–91.7)	83.3 (66.7–91.7)	8.3	11.1	−3.319	0.001
Cognitive function	190	80.0 (60.0–90.0)	85.0 (65.0–95.0)	5.0	6.3	−3.603	<0.001
Health distress	190	62.5 (43.8–80.0)	80.0 (60.0–90.0)	17.5	28.0	−7.446	<0.001
Sexual function	166	79.2 (58.4–91.7)	83.4 (58.3–100.0)	4.2	5.3	−0.590	0.555
Overall quality of life	195	73.4 (60.0–81.7)	81.7 (68.4–86.7)	8.3	11.3	−5.433	<0.001
Change in health	194	50.0 (25.0–50.0)	75.0 (50.0–75.0)	25.0	50.0	−7.017	<0.001
Satisfaction with sexual function	168	50.0 (25.0–75.0)	75.0 (50.0–100.0)	25.0	50.0	−2.130	0.033
Physical health composite	149	63.7 (48.6–77.8)	75.50 (56.4–84.9)	11.8	18.6	−5.408	<0.001
Mental health composite	178	72.9 (51.9–85.1)	81.5 (68.7–90.3)	8.6	11.8	−5.697	<0.001

Differences analysed using Wilcoxon Signed Rank test

*n* Number of participants, *IQR* interquartile range

### 5-year time-point

Similar changes were detected at the five year time-point (Table 4). Of those reaching the 5-year time-point (*n* = 165), surveys were completed by 96 participants, for a 58% response rate. Improvements were observed in 11 of the 14 MSQOL domains, of which nine were statistically significant. Three domains detected no change over time including change in health, sexual function and satisfaction with sexual function. The latter two domains were not statistically significant, nor were physical health or pain scale scores. Akin to 1 year, despite no median change over time, significant differences were still detected by the Wilcoxon Signed Ranks test for change in health (*p* = 0.009). The biggest improvements after 5 years were observed in role limitations due to physical problems with a 37.5 point increase (100% median difference; *p* = 0.001), followed by a 17.5-point median improvement in health distress (*p* < 0.001). Both the mental health and physical health composites were statistically significant and improved over time with a percentage median difference of 22.8 and 17.8% respectively, over 5 years, with an overall improvement in quality of life of 19.5% (*p* < 0.001) (Table 4; Fig. 1).

**Table 4:** Comparison of domain and composite scores at baseline and 5-year time-points

Domain	*n*	Baseline	5 year	Domain point difference	% median difference	*Z* score	*p* value
		Median (IQR)					
Physical health	96	75.0 (46.3–95.0)	82.5 (40.0–95.0)	7.5	10.0	−1.122	0.262
Role limitations-physical	96	37.5 (0.0–100.0)	75.0 (6.3–100.0)	37.5	100.0	−3.921	<0.001
Role limitations-emotional	92	83.3 (0.0–100.0)	100.0 (66.7–100.0)	16.7	20.0	−3.967	<0.001
Pain	95	85.0 (70.00–100.00)	93.33 (71.7–00.0)	8.3	9.8	−0.669	0.503
Emotional well-being	96	72.0 (56.0–84.0)	80.0 (68.0–88.0)	8.0	11.1	−4.328	<0.001
Energy	96	48.0 (29.0–64.0)	56.0 (40.0–72.0)	8.0	16.7	−3.735	<0.001
Health perceptions	95	60.0 (45.0–75.0)	75.0 (55.0–85.0)	15.0	25.0	−4.334	<0.001
Social function	90	75.0 (58.3–91.7)	83.3 (66.7–100.0)	8.3	11.1	−2.875	0.004
Cognitive function	96	80.0 (56.3–90.0)	85.0 (65.0–95.0)	5.0	6.3	−2.023	0.043
Health distress	94	62.5 (35.0-80.0)	80.0 (63.8–95.0)	17.5	28.0	−6.100	<0.001
Sexual function	84	75.0 (58.3–91.7)	75.0 (50.0-91.7)	0.0	0.0	−0.167	0.868
Overall QOL	95	68.4 (58.4–81.7)	81.7 (63.4–90.0)	13.3	19.5	−3.603	<0.001
Change in health	94	50.0 (25.0–50.0)	50.0 (50.0–75.0)	0.0	0.0	−2.599	0.009
Satisfaction with sexual function	87	50.0 (25.0–75.0)	50.0 (25.0–75.0)	0.0	0.0	−0.417	0.667
Physical health composite	78	64.5 (47.4–78.4)	75.9 (56.8–87.7)	11.4	17.8	−3.887	<0.001
Mental health composite	89	67.4 (50.2–85.1)	82.8 (72.1–90.1)	15.4	22.8	−4.924	<0.001

Differences analysed using Wilcoxon Signed Rank test

*n* Number of participants, *IQR* interquartile range

## Discussion

Our study, the first of its kind, indicates a significant persistent improvement in short- and medium-term HRQOL for people with MS after attending a live-in educational retreat. 1 and 5 years after attendance, most of the MSQOL-54 domains demonstrated significant improvements, with no domain detecting a decline in self-reported HRQOL. This applied to both mental and physical health composites, which continued to improve over the 5-year time frame. As MS is known to result in deteriorating quality of life and increasing disability over time, these results are remarkable.

MS can have a profound impact on a person’s quality of life and in the absence of a cure, maintenance of function and quality of life are the focus of treatment. Clinical outcome measures of relapse rate and disability are insufficient alone to assess the impact of disease, thus a more broad measurement tool is required [25, 26]. HRQOL is a multifaceted concept that incorporates the symptom-related impact of disease as well as psychosocial aspects such as fatigue, emotional well-being or participation [27]. A quality of life tool can be used to assess health disparities within a population or the effectiveness of interventions. Self-reported data in MS cohorts have been found to be a reliable measure of patient outcomes [28, 29]. In our study, due to the varied level of participant disability, both hardcopy and online questionnaire formats were offered to ease facilitation of survey completion. The online format was found to be the preferred method of most participants.

The mental health composite scores demonstrated clinically and statistically significant improvements at both 1 and 5-year time points. This is important because prevalence rates of depression and anxiety are high in patients with MS and can affect the way a patient copes with the limitations of the disease [30]. Social isolation and a lack of social support can further exacerbate psychological decline, leading to poorer quality of life [30, 31]. “Invisible” symptoms of MS (depression, anxiety, fatigue and pain) can be more difficult to assess clinically but are thought to have a greater impact on health distress [32]. More concerning, is evidence demonstrating the effect that poor psychological health can have on disease progression. A meta-analysis [33] of the relationship between stressful life events and MS exacerbations found a clinically meaningful effect size. This supports earlier work showing an increased risk of brain lesions 8 weeks after the occurrence of stress, with preoccupation and coping as moderators of this effect [34]. The stress reduction techniques and meditation taught at the retreats may assist participants in coping with high levels of stress and drive the considerable improvements in the mental health composite scores. Such interventions can easily be incorporated into a management plan for dealing with adjustment issues and stress and may also slow disease progression.

People with MS constitute a heterogeneous group with diverse needs and modes of adaptation in coping with the limitations of the illness [35]. In contrast with physicians, people with MS may view physical disability as less important than other quality of life dimensions such as emotional well-being and vitality [36]. The process of patient-centred care involves engaging individuals and encouraging them to share in the decision-making role to create a disease management plan that incorporates their physical, psychological and socio-cultural needs [37]. Shifting the paradigm of MS care to a patient-centred approach has the potential to improve patient outcomes and satisfaction with the provider’s service [5]. It is thought that mastery, or the degree to which people feel they are in control of their illness, plays an important role in health status. Patients with higher levels of self-efficacy and empowerment report lower rates of depression [38] and are more likely to effectively self manage their illness through the appropriate utilisation of services and treatment adherence [39]. The facilitators have recognised tailoring patient-centred care and fostering empowerment as important approaches in the design and operation of our MS programme. This might help explain why patients report lower rates of health distress and improved emotional well-being 5 years after the retreat.

Despite improvements in the sexual health domains at the 1-year time-point, no change was observed at 5 years. Sexual dysfunction is common in MS with 40–85% of women and 50–90% of men being adversely affected [40]. One study found a significant negative correlation between sexual dysfunction and nearly all MSQOL-54 domains [41]. It presents a significant burden for self-esteem and relationships but unfortunately, this problem is insufficiently addressed by both the person with MS and the physician who are often reluctant to discuss it [42]. Although there was no worsening of sexual function, perhaps the programme could be adapted to more openly discuss some of the potential solutions to this prevailing issue. Pain is also a difficult management issue, with no really effective pharmacological agents available, and no significant change in pain detected at the 5-year point following our programme.

The global improvements in health status observed over the 5-year period are considerably better than one would expect, given the usually progressive, degenerative nature of the illness. While it is difficult to make comparisons with other studies as few have observed patients for more than 2 years, in general, studies measuring HRQOL over time report a predominant decline in the health status of the participants [43–46]. One study [45], which measured MSQOL-54 over a 5-year period documented no change, or a worsening in 11 of the 14 health domains. This contrasts dramatically with our study. A wellness programme for women in the United States [47] saw some improvements in pain and mental health scales, however, the follow-up concluded only 8-months after the intervention.

### Limitations

A longitudinal cohort study is the appropriate method to assess outcomes in MS, which is usually progressive and varies for individuals over time. The absence of a control group, and declining response rates over time, however, decrease the strength of the evidence. With more than half the participants returning 5-year follow-up data, and no domain worsening over that period, it is unlikely, however, that we are simply observing a benign MS group, who are thought to comprise only a small fraction of people with MS, and whose quality of life and function are documented to deteriorate over time [48].

It is difficult to know which aspects of the programme had the greatest impact on self-reported quality of life. The favourable results could be viewed as being related to the participants’ altered perception of quality of life over time, as opposed to a tangible change, or that they simply learned to cope with the limitations of the disease. This is unlikely though, because most studies have shown deterioration in HRQOL over time using the MSQOL-54. Additionally, there may have been participation bias, although there is no clear reason why those not providing consent to participate would have had a different course than those participating. Similarly, the follow-up results may, in part, be attributed to responder bias. However, comparison of the baseline overall quality of life domain showed no significant difference between participants who only completed a baseline questionnaire and those who continued to contribute data at the 1 or 5 year time-points.

We do not currently know the degree to which participants adhered to each individual lifestyle intervention long-term. Future research on this cohort will include measures of programme adherence. Additionally, it is unknown to what degree pharmaceutical intervention had an impact on quality of life, both in terms of reduced relapse rates and worsening of HRQOL due to side effects. We can, however, anecdotally report that many of the participants were not taking immune-modulating therapies.

MS diagnosis was self-reported by participants. The few people reporting neurological disorders other than MS were permitted to take part in the programme but excluded from analysis. Additionally, it is unknown which type of MS the participants had been diagnosed with and whether they were representative of the average distribution in the general MS population.

## Conclusion

This study adds to the evidence supporting the use of integrated non-drug therapies for people with MS. Lifestyle modification should form part of any comprehensive chronic disease management plan, and given that there are likely to be other health benefits associated with this approach, there is considerable merit in offering this to people with MS. In addition to the benefits patients should experience, there are also likely to be reduced healthcare costs and improved satisfaction with the health service. The promotion of lifestyle intervention is not in competition with the use of pharmacological therapies; both should be considered for their potential benefits and risks, with decisions tailored to the needs of the individual. Trials assessing the impact of lifestyle modification long-term are methodologically complex and there are fewer financial incentives to invest in such research. Regardless, this study adds to the evidence supporting the use of a range of lifestyle interventions for people with MS.
